# A Thorn in a Haystack: A Rare Case of Septic Arthritis

**DOI:** 10.7759/cureus.20519

**Published:** 2021-12-19

**Authors:** Marta Leal-dos-Santos, José N Ferreira, David Peres, Carlos Palos

**Affiliations:** 1 Infectious Diseases Department, Hospital de Curry Cabral-Centro Hospitalar Universitário de Lisboa Central, Lisbon, PRT; 2 Orthopaedics Department, Hospital Beatriz Ângelo, Loures, PRT; 3 Infection and Antibiotic Resistance Prevention and Control Unit, Hospital Pedro Hispano-Unidade Local de Saúde Matosinhos, Matosinhos, PRT; 4 Infection and Antibiotic Resistance Prevention and Control Unit, Hospital Beatriz Ângelo, Loures, PRT

**Keywords:** pantoea agglomerans, foreign body, gram negative, septic arthritis, thorn injurie

## Abstract

*Pantoea agglomerans* is a facultative anaerobe and environmental bacterium that could be a cause of opportunistic human infections, especially in wound infection with plant material. ﻿Arthritis or synovitis secondary to penetrating plant thorn injuries is not frequently reported. We present the case of a 35-year-old otherwise healthy male with a bramble thorn penetrating injury of the left knee. *P. agglomerans* was isolated from the synovial fluid. The patient was treated with amoxicillin/clavulanate according to sensitivity testing. This case highlights the importance of precise and thorough medical history, especially for less common presentations, as well as source control.

## Introduction

*Pantoea agglomerans* is a facultative anaerobe and environmental bacterium of the family *Enterobacterales* found in plants, the earth, and water, and occasionally in wounds of animals [1,2]. ﻿*P. agglomerans*, though not an obligate infectious agent in humans, could be a cause of opportunistic human infections, especially in wound infection with plant material [3]. Arthritis or synovitis secondary to penetrating plant thorn injuries is not frequently reported. Historically, it is considered aseptic and treated with removal of the intra-articular foreign body and affected synovial lining [4]. To our knowledge, there have been only eight case reports in the literature (11 patients), which shows that this rare type of arthritis or synovitis can be difficult to diagnose because of an insidious onset after an apparently trivial injury.

## Case presentation

**Figure 1:**
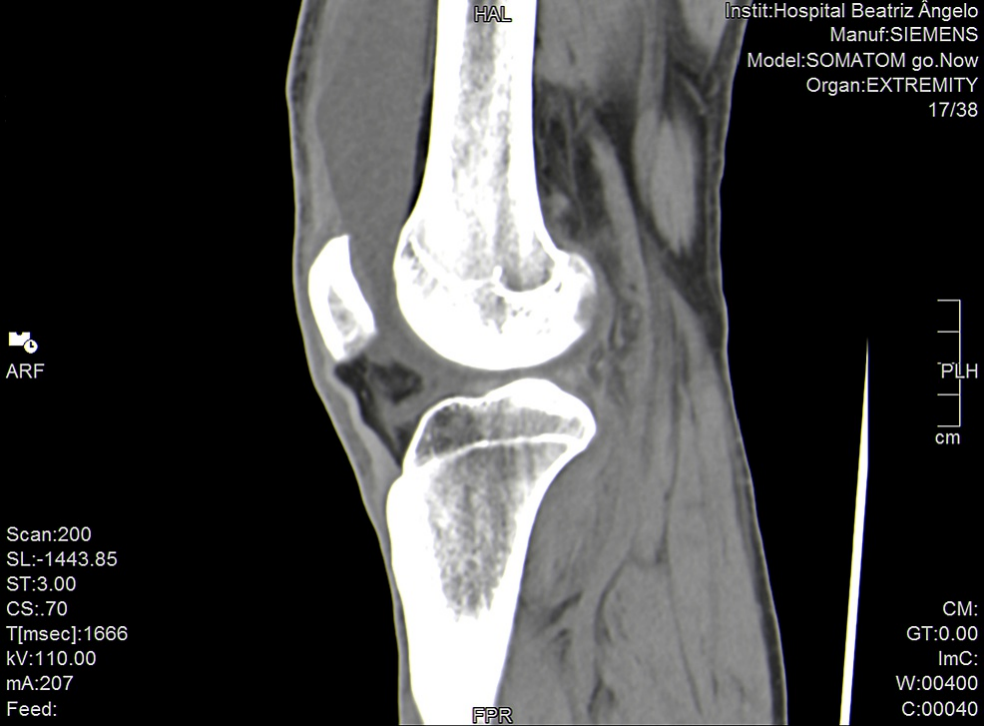
Increased articular liquid extending to subquadricipital space (sagital view).

**Figure 2:**
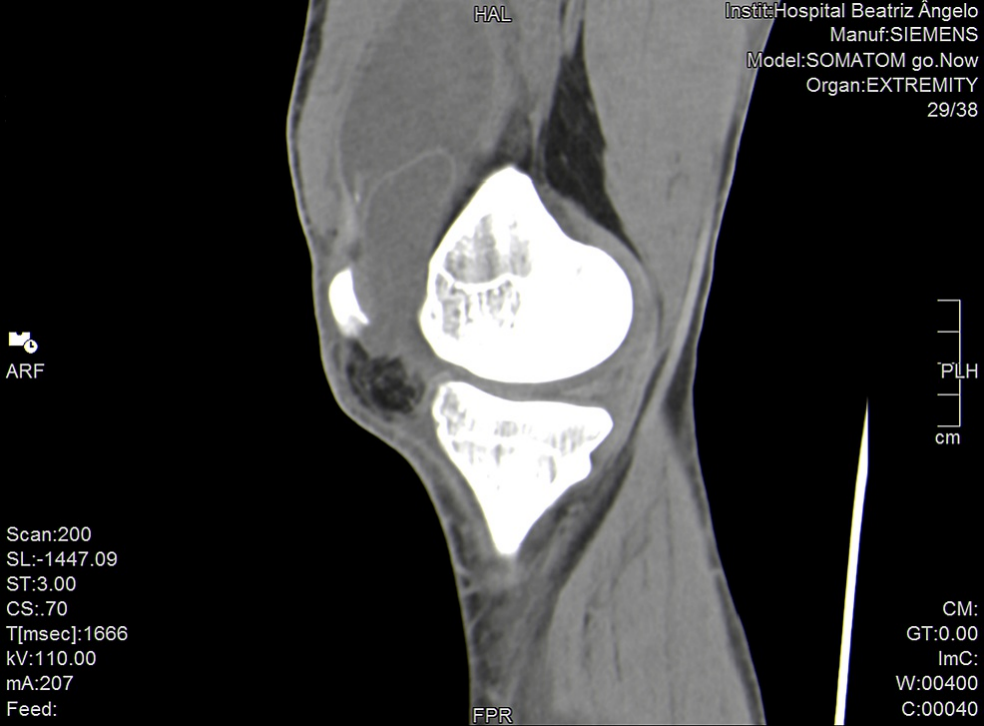
Subquadricipital space with septae forming two loci filled with fluid (sagital view).

**Figure 3:**
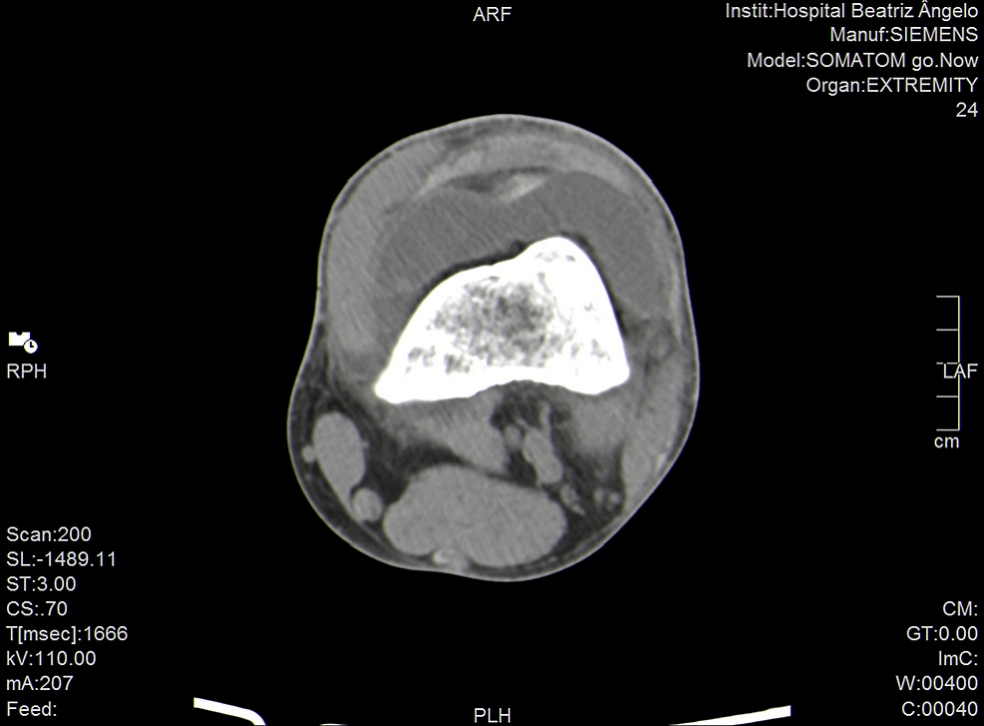
Distal portion of femur, showing intra-articular fluid (axis view).

We present the case of a 35-year-old otherwise healthy male, including no immunosuppressant conditions or medications, with a bramble thorn penetrating injury of the left knee. The patient presented for two occasions to the emergency department, one week apart. Firstly, the patient complained of pain and swelling of the left knee, with limited flexion and inability to walk, and was started on amoxicillin/clavulanate and non-steroid anti-inflammatory drugs. On a second occasion, he presented with persistent pain, swelling, and reduced range of motion but was able to walk with aid and apparently with the presence of liquid and was admitted to the hospital. On physical examination and on both occasions, he was afebrile, but on the second visit to the emergency room, he showed a tense, swollen knee joint with redness and tenderness. A millimetric entry point was identified on the anterolateral surface of the knee, but no foreign body was visible. Septic arthritis was considered, and arthrocentesis was performed, revealing a turbid yellow fluid with 76,720 leukocytes/mL with 98% polymorphonuclears. Gram stain was negative. Blood tests showed an elevated C-reactive protein of 17.6 mg/dL (normal value <0.5 mg/dL), with leucocytosis (11.88 × 10^9^/L, normal range 4.00-10.00 × 10^9^/L) and no neutrophilia. Computed tomography showed a significant amount of fluid with subquadricipital internal septa (Figures 1-3).

Upon admission, an arthroscopy and lavage were performed, and fluid was sent for culture and sensitivity testing. No thorn was retrieved, hence no identification was possible. Empirical antibiotic therapy was started with flucloxacillin, pending microbiological results. On the third day of admission, *P. agglomerans* was isolated from the synovial fluid, and after consulting with the antibiotic stewardship team antibiotics were changed back to amoxicillin/clavulanate according to sensitivity testing (Table 1).

**Table 1:** Antibiotic susceptibilities.

Antibiotic susceptibilities	P. agglomerans
Amoxicillin/slavulanate	S
Ciprofloxacin	S
Gentamicin	S
Cotrimoxazol	S
Ampicillin	R
Piperacillin/tazobactam	R

The patient showed clinical improvement and inflammatory markers became negative and was discharged after 10 days.

## Discussion

Monoarthritis of the knee is a common diagnosis in the emergency room setting with a broad variety of possible aetiologies [5]. Traumatic aetiologies are the most frequent, nevertheless, infectious diseases should be suspected in some specific cases [5]. Plant thorn-related arthritis is well described but may be overlooked as a differential diagnosis of monoarticular inflammatory disease.

Thorn arthritis/synovitis are classically considered aseptic with three underlying physiopathological mechanisms: foreign body reaction, toxic substance reaction, or immunological reaction to a foreign body [6]. The common link is inflammation with a certain number of cases being infected [4]. Like usually described in non-infectious and infectious plant thorn arthritis/synovitis, our patient presented without fever and with a tender joint that yielded a turbid fluid. Classically, retrieved joint fluid is clearly inflammatory predominantly polymorphonuclear and neutrophilic, rarely lymphocytic [4,6]. Similarly, our patient had a fluid with a high leukocyte count and predominance of polymorphonuclear cells.

Diagnosis is frequently delayed for months or even years, particularly when the initial trauma has been forgotten or if that hypothesis has not been considered upfront [6]. Differently from what is reported in the literature, where most cases present in a subacute/chronic fashion [4-6], in the present case, we had a patient with an acute presentation favouring the link between the thorn and the clinical picture. Additionally, arthritis secondary to plant thorn prick is an established medical condition occurring, mostly in children [5] and not adults-like our case. In our case, no thorn was retrieved from arthroscopy, hence a clinical presumptive diagnosis was made since there was a confirmed history of a bramble thorn penetrating injury that the patient remembered accurately and the identification of a thorn-related pathogen like *P. agglomerans*.

The possibility of septic arthritis caused by a slow-growing microorganism is the main concern in the differential diagnosis (*Mycobacteria* or *Enterobacterales*). In most cases, the cultural examination is sterile, however, rarely, it grows gram-negative bacteria, more commonly *P. agglomerans* [6]. In our case, microbiological results were considerably faster than expected, which might be attributable to faster microbiological detection methods and/or clinical suspicion since trauma with a thorn was taken into account from the beginning.

*P. agglomerans* antimicrobial sensitivity is variable, it is frequently resistant to β-lactams [6]. *P. agglomerans* and other *Enterobacterales* may produce β-lactamase and are not always covered by the conventional empirical antibiotic treatment schemes for septic arthritis [1]. In previous studies, *P. agglomerans* has shown sensitivity to amikacin, gentamicin, carbapenems, cotrimoxazole, ampicillin/sulbactam, cephalosporines, and quinolones [6]. However, in our case, we had confirmation of susceptibility to amoxicillin/clavulanate hence the choice to change back from flucloxacillin. In line with the previously described case series [1], antibiotic therapy alone was ineffective in this case, as evidenced by the patient's return to the emergency room with a tense joint after starting antibiotics, necessitating an arthroscopy and lavage.

## Conclusions

This case highlights the importance of precise and thorough medical history, especially for less common presentations. It also shows how source control is a cornerstone of septic arthritis and the importance of microbiology results for adequate antibiotic therapy.

As with other infections, antibiotic therapy should be initiated as soon as possible. Empirical therapy should begin as soon as fluids for culture are harvested and should be adjusted, according to microbiological reports. If source control cannot be achieved and antibiotic treatment fails, medical history should be clarified while keeping a high suspicion for an occult foreign body. Early open debridement of the joint with an extensive search for the thorn seems justified when the culture of synovial fluid yields *P. agglomerans*.
